# Micromechanical Prediction of Elastic Properties of Unidirectional Glass and Carbon Fiber-Reinforced Epoxy Composites Using the Halpin–Tsai Model

**DOI:** 10.3390/polym18070822

**Published:** 2026-03-27

**Authors:** Sahnoun Zengah, Rabeh Slimani, Abdelghani Baltach, Ali Taghezout, Ali Benhamena, Dursun Murat Sekban, Ecren Uzun Yaylacı, Murat Yaylacı

**Affiliations:** 1 Department of Mechanical Engineering, University of Mustapha Stambouli, Mascara 29000, Algeria; s.zengah@univ-mascara.dz (S.Z.); r.slimani@univ-mascara.dz (R.S.); ali_taghezout@yahoo.fr (A.T.); ali_benhamena@yahoo.fr (A.B.); 2 Mechanics of Materials, Energy and Environment Laboratory (L2M2E), University of Mustapha Stambouli, Mascara 29000, Algeria; abdelghani.baltach@univ-tiaret.dz; 3 Department of Mechanical Engineering, University of Ibn Khaldoun, Tiaret 29000, Algeria; 4 Department of Marine Engineering Operations, Karadeniz Technical University, 61080 Trabzon, Türkiye; msekban@ktu.edu.tr; 5 Trabzon Teknokent, WMS Engineering Services Industry Trade Limited Company, 61080 Trabzon, Türkiye; 6 Faculty of Fisheries, Recep Tayyip Erdogan University, 53100 Rize, Türkiye; ecren.uzunyaylaci@erdogan.edu.tr; 7 Department of Civil Engineering, Recep Tayyip Erdogan University, 53100 Rize, Türkiye; 8 Turgut Kıran Maritime Faculty, Recep Tayyip Erdogan University, 53900 Rize, Türkiye; 9 Dijitalpark Teknokent, Murat Yaylacı-Luzeri R&D Engineering Company, 53100 Rize, Türkiye

**Keywords:** micromechanics, fiber-reinforced composites, elastic behavior, Halpin–Tsai model

## Abstract

This study presents a calibrated analytical micromechanical framework for predicting the linear elastic behavior of unidirectional glass fiber/epoxy and carbon fiber/epoxy composites over a wide range of fiber volume fractions. The approach combines the classical rule of mixtures for the longitudinal Young’s modulus with the semi empirical Halpin–Tsai equations to estimate the transverse Young’s modulus and the in-plane shear modulus. The framework is specifically formulated to support durability-oriented composite design through rapid and physically consistent estimation of elastic properties governing load transfer and stress distribution. Material parameters, including fiber and matrix Young’s moduli (Ef, Em), shear moduli (Gf, Gm), Poisson’s ratios (νf, νm), and fiber volume fraction (Vf up to 0.80), are taken from established material property databases and implemented within a literature-informed modeling scheme. To preserve physical realism at high fiber contents, a shear correction factor is introduced for Vf > 0.50 to account for microstructural interaction and fiber clustering effects. The predicted effective elastic constants (E_1_, E_2_, G_12_, ν_12_) exhibit consistent and physically meaningful trends across the full fiber volume fraction range. The model predictions were evaluated against trends widely reported in the composite micromechanics literature, and the results showed overall agreement in the nonlinear reduction in stiffness gains at elevated fiber volume fractions. Comparative results indicate that carbon fiber/epoxy composites achieve up to approximately 30% higher stiffness than glass fiber/epoxy systems at equivalent fiber contents, reflecting the influence of stiffness contrast on composite response. The analysis further indicates that stiffness saturation begins approximately in the Vf = 0.60–0.70 range, where the incremental gains in E2 and G12 become noticeably smaller for both composite systems. This behavior provides design-relevant guidance by showing that, beyond this range, further increases in fiber content may offer limited stiffness improvement relative to the associated manufacturing complexity. Overall, the calibrated Halpin–Tsai methodology offers a practical and computationally efficient tool for preliminary evaluation and design-stage optimization of the elastic performance of high-performance composite structures.

## 1. Introduction

The long-term performance of composite structures is governed not only by the intrinsic properties of their constituent materials but also by the quality of manufacturing and the accuracy of property prediction at the design stage. In structural applications, reliable estimation of elastic constants is essential because stiffness controls load transfer, stress distribution, deformation compatibility, and the initiation of local damage. Therefore, the design of polymer matrix composites requires a consistent relationship between constituent selection, preparation technology, and micromechanical prediction of the effective composite response. In polymer matrix composites, durability is inherently multifactorial and depends on environmental exposure, cyclic mechanical loading, fabrication quality, and the intrinsic properties of fibers and matrices [1,2,3,4]. Under controlled operating conditions, glass and carbon fiber-reinforced epoxy laminates frequently achieve service lives exceeding ten years; however, aggressive environments involving ultraviolet (UV) radiation, moisture ingress, thermal cycling, and high-frequency fatigue loading necessitate a holistic durability-driven design approach. In this respect, accurate prediction of elastic constants, particularly matrix dominated properties such as E_2_ and G_12_, provides a useful basis for understanding stress transfer, interfacial response, and damage initiation in durability-sensitive composite systems.

Environmental degradation is one of the primary mechanisms governing long-term performance. UV radiation induces photo-oxidative reactions in the polymer matrix, resulting in chain scission, matrix embrittlement, and progressive degradation of the fiber–matrix interface. Shi et al. [4] reported compressive strength reductions of ~23% in carbon fiber-reinforced polymer (CFRP) laminates after prolonged UV exposure. Moisture absorption further accelerates damage by plasticizing the matrix and weakening interfacial adhesion, thereby increasing susceptibility to fatigue crack initiation [5,6,7,8].

Mechanical fatigue is another dominant factor influencing composite durability. Manufacturing-induced defects such as micro voids, fiber waviness, and resin-rich zones act as stress concentrators that promote early crack initiation and accelerate damage propagation under cyclic loading. Hetrick et al. [9] demonstrated that increased void content significantly reduces tensile strength and fatigue life in fiber-reinforced composites. These findings highlight the importance of stringent manufacturing process control.

Manufacturing parameters are equally critical because they directly influence the microstructural variables that govern the effective elastic properties of the composite. Cure temperature and pressure, resin viscosity, fiber placement quality, and consolidation conditions affect void content, fiber alignment, matrix continuity, and the quality of fiber–matrix interfacial bonding. As a result, preparation technology has a quantitative influence on design development: process-dependent changes in fiber volume fraction, defect content, and local fiber distribution modify the engineering constants used in analytical design models and finite element analyses. Recent studies have emphasized this process–structure–property relationship and shown that manufacturing route and material tailoring can significantly affect stiffness retention, fatigue resistance, and long-term mechanical stability in FRP systems [10,11,12,13,14].

Durability assessment is structured around key metrics such as service life, useful life, and structural robustness. These metrics provide a framework for evaluating composites across aerospace, civil infrastructure, and renewable energy applications. By integrating environmental aging tests, fatigue characterization, and process optimization, composites can be engineered to satisfy stringent durability requirements while minimizing lifecycle costs [15,16]. However, such durability-oriented design ultimately relies on accurate estimation of the elastic properties that define the initial mechanical state of the composite system.

Accordingly, micromechanical modeling approaches based on mixture rules and Halpin–Tsai formulations have been widely adopted to predict the effective elastic properties of composite materials and to support durability and performance-oriented structural design [17,18,19,20]. In addition to these classical approaches, recent studies have also employed modified Halpin–Tsai relations, Mori–Tanaka homogenization, and representative volume element (RVE)-based methods to improve the prediction of composite stiffness and to better capture the influence of microstructural architecture over a broad range of fiber volume fractions [21,22]. Such homogenization approaches are particularly useful for describing the influence of inclusion interaction, phase distribution, and local stress transfer mechanisms in fiber-reinforced polymer composites. These developments confirm that micromechanical property prediction remains a central tool for linking constituent-level material selection and manufacturing characteristics to structural design.

Originally introduced by Halpin and Kardos [21,23], the Halpin–Tsai micromechanical model provides a semi-empirical interpolation framework for estimating the transverse Young’s modulus (E_2_) and the in-plane shear modulus (G_12_) of fiber-reinforced composites as a function of the fiber volume fraction. In this formulation, the stiffness contrast parameter η, which quantifies the relative difference between the fiber and matrix rigidity, is defined as follows:

η = (Mf/Mm − 1)/(Mf/Mm + ξ)(1)

where Mf denotes the modulus of the fibers (either their Young’s modulus when calculating E2 or their shear modulus for G12) and Mm denotes the corresponding modulus of the polymer matrix. Meanwhile, the geometric factor ξ accounts for fiber shape and spatial arrangement, and the fiber volume fraction Vf represents the volumetric proportion of fibers in the composite.

Consequently, the effective composite modulus M (interpreted as either E_2_ or G12 depending on context) follows from

M = Mm (1 + ηξVf)/(1 − ηVf)(2)

so that as ξ tends toward zero, the prediction collapses to M = Mm (the matrix dominated, series connected limit), whereas for very large ξ, the model converges on the rule of mixtures upper bound

M = Vf Mf + (1 − Vf) Mm.(3)

Typically, values of ξ = 2 for E_2_ and ξ = 1 for G_12_ are adopted. However, at elevated fiber volume fractions (Vf > 0.50), local microstructural effects such as fiber clustering, non-uniform resin distribution, and reduced matrix continuity may cause the standard Halpin–Tsai formulation to overestimate the in-plane shear response. For this reason, an empirical shear-correction factor was introduced in the present study only for G_12_ in the high volume fraction regime. This correction is not introduced as a new constitutive model but rather as a calibrated extension intended to improve the physical realism of analytical predictions in a range where standard semi-empirical relations may become overly optimistic. The explicit form, calibration basis, and physical justification of this correction are provided in Section 2.

Moreover, to furnish a complete set of engineering constants, the model is frequently coupled with simple mixture rules for the longitudinal modulus and Poisson’s ratio. In particular, the longitudinal Young’s modulus E1 is given by

E1 = Vf Ef + (1 − Vf) Em(4)

where Ef and Em are the Young’s moduli of fiber and matrix, respectively, and the effective Poisson’s ratio ν12 follows

ν12 = Vf νf + (1 − Vf) νm(5)

with νf and νm signifying the fiber and matrix Poisson’s ratios. Consequently, this hybrid approach yields the full tensor of engineering constants (E1, E2, G12, ν12), thereby underpinning both preliminary composite design and more detailed finite element analyses. Accordingly, E1 and ν12 are obtained directly from the mixture rules, whereas E2 and G12 are obtained from the Halpin–Tsai-based calculation procedure described above.

Despite the availability of more advanced homogenization strategies, simplified semi-empirical models remain highly valuable in preliminary engineering design because they provide rapid, transparent, and computationally efficient estimates of composite behavior. From a practical standpoint, there remains a need for an analytical framework that enables direct comparison of glass fiber/epoxy and carbon fiber/epoxy systems on a common basis while also incorporating a physically motivated adjustment for the high fiber volume regime.

In this context, the present study develops a calibrated and comparative micromechanical framework combining classical mixture rules with a calibrated Halpin–Tsai formulation to systematically evaluate the elastic properties of unidirectional glass and carbon fiber-reinforced epoxy composites over a wide range of fiber volume fractions (Vf up to 0.80). Rather than proposing a fundamentally new micromechanical theory, this study aims to provide a design-oriented extension of established analytical relations for consistent comparison, parametric interpretation, and preliminary material screening. The main contribution of this study is the comparative and design-oriented assessment of the complete engineering constant set for both composite systems within a common framework, together with a high volume fraction correction applied to the in-plane shear response. By quantifying the influence of fiber type, stiffness contrast, and fiber volume fraction on E1, E2, G12, and ν12, this study provides practical insight for the preliminary design and material selection of high-performance composite structures. In practical terms, the proposed model is intended for preliminary composite design, comparative material selection, and parametric evaluation of unidirectional glass and carbon fiber/epoxy systems, particularly within the fiber volume fraction range commonly achievable in manufacturing. Accordingly, the scope of the present work is limited to micromechanical prediction of elastic behavior and its relevance to composite design. The contribution of this study lies in integrating established analytical expressions within a single calibrated framework and in examining their response across a broad fiber volume fraction range in a manner that is consistent with trends reported in the composite micromechanics literature.

## 2. Methodology

### 2.1. Materials Used

In this study, the careful selection of constituent materials underpins both the mechanical performance and long-term durability of the resulting fiber-reinforced composites. By integrating high strength reinforcements with a robust polymer matrix, we establish a foundation that supports subsequent micromechanical modeling and literature-based assessment of the predicted elastic response.

Firstly, high-tensile “R” glass fiber was selected for its exceptional reinforcement capabilities. This fiber, often classified as R-glass or S-glass in European standards, offers a Young’s modulus of 86 GPa, a tensile strength of 3200 MPa, and a density of 2500 kg/m^3^. Its outstanding stiffness-to-weight ratio makes it ideal for applications where rigidity and load-bearing capacity are critical [21]. In the present micromechanical framework, the shear modulus of the glass fiber was not required as an independent input parameter because the transverse and shear properties were evaluated using Halpin–Tsai relations based primarily on the stiffness contrast between the fiber and the matrix. Therefore, the glass fiber shear modulus was not explicitly listed in Table 1.

Secondly, high-resilience (HR) carbon fiber was employed to explore the effects of increased stiffness contrast within the composite. Characterized by a Young’s modulus of 230 GPa, a shear modulus of 50 GPa, and a density of 1750 kg/m^3^, this fiber combines remarkable rigidity with sufficient flexibility to resist fracture under bending loads. The corresponding Poisson’s ratio (ν) was taken as 0.30. With a typical carbon content of 90%, HR carbon fiber also delivers superior fatigue performance, making it suitable for dynamic applications such as sporting equipment and aerospace components [24,25,26,27,28]. Recent studies show that HR carbon fiber composites offer improved mechanical stability under aging and environmental conditions [29,30,31]. Poisson’s ratio of the carbon fiber was taken as 0.30, which falls within the typical engineering range reported for carbon fiber-reinforced polymer systems and is frequently adopted in micromechanical estimations. This value was used to maintain consistency in the Halpin–Tsai formulation for predicting the transverse elastic response of the composite system.

Finally, a two-component epoxy resin system served as the polymer matrix. The resin (Young’s modulus of 3.4 GPa, Poisson’s ratio of 0.2, and density of 1200 kg/m^3^) was mixed with its corresponding hardener immediately prior to processing to ensure optimal cross-link density and interfacial adhesion. This matrix formulation facilitates efficient load transfer between fibers and resin, thereby enhancing the composite’s overall mechanical integrity. All constituent material properties reported in Table 1 were selected from representative manufacturer datasheets and commonly reported literature values for glass fiber, carbon fiber, and epoxy systems. These parameters were used as input values for the micromechanical calculations based on the classical rule of mixtures and the Halpin–Tsai relations. The symbols E, G, and ν denote Young’s modulus, shear modulus, and Poisson’s ratio, respectively, while the corresponding units are GPa for moduli, MPa for tensile strength, and kg/m^3^ for density. The key physical and mechanical properties of the selected fibers and epoxy matrix used in the micromechanical modeling are summarized in Table 1.

### 2.2. Program Structure and Methodology

The computational framework developed in this study provides a high-performance environment for the simulation of composite mechanical behavior. The algorithm combines classical analytical mixture rules with semi-empirical Halpin–Tsai relations to predict the elastic response of unidirectional fiber-reinforced composites [20]. This integrated approach enables rapid evaluation of longitudinal, transverse, and shear properties while maintaining full control over calibration parameters and sensitivity analysis.

All constituent material properties, including Young’s moduli, shear moduli, and Poisson’s ratios of both fibers and matrix, are first imported from external data sources. The fiber volume fraction (Vf) is defined over a continuous range from 0 to 0.80 using fine increments. To preserve the validity of the semi-empirical framework, a constraint is applied to the product of the stiffness-contrast parameter and the fiber fraction, preventing non-physical predictions. More specifically, the calculation procedure starts with the definition of the constituent inputs (Ef, Em, Gf, Gm, νf, and νm), after which the code loops over the prescribed Vf range and computes the corresponding engineering constants at each increment.

The evaluation begins with the prediction of the longitudinal modulus under the classical isostrain assumption. The rule of mixtures is used to determine the axial Young’s modulus (E_1_) and the corresponding Poisson’s ratio. Assuming perfect fiber–matrix adhesion, this step establishes a reference solution against which transverse and shear predictions are compared. At each fiber volume fraction, E_1_ is first calculated from the rule of mixtures, and ν12 is then obtained using the same constituent-weighted linear combination.

A dedicated computational module is then employed to evaluate the Halpin–Tsai relations. Stiffness-contrast parameters are computed separately for the transverse modulus (E_2_) and the in-plane shear modulus (G_12_) [23]. The geometric factor associated with each deformation mode is used to interpolate between matrix-dominated and fiber-dominated limits [32]. Encapsulation of these operations within an independent routine ensures modularity and facilitates adaptation to varying fiber aspect ratios or alternative composite systems [33]. In practical terms, the algorithm first evaluates η for E_2_ by using the Young’s modulus ratio of fiber to matrix and then evaluates η for G_12_ by using the corresponding shear modulus ratio. These η values are inserted into the Halpin–Tsai expression together with the selected geometric factors to obtain E_2_ and G_12_ for the same Vf level.

To incorporate microstructural stiffening mechanisms at high fiber contents, an empirical correction is applied when the volume fraction exceeds 0.50. In the present implementation, the corrected in-plane shear modulus is expressed as

G_12_,corr = G_12_,HT·f_s_(Vf), for Vf > 0.50

where G_12_,HT is the standard Halpin–Tsai prediction and f_s_(Vf) is the empirical shear-correction factor. In this study, f_s_(Vf) was introduced as a decreasing linear adjustment in the high volume fraction regime, defined as

f_s_(Vf) = 1 − α(Vf − 0.50), for Vf > 0.50 and f_s_(Vf) = 1, for Vf ≤ 0.50,

where α = 0.30 is an empirical shear-degradation coefficient adopted in the present study, consistent with literature-reported deviations observed in high volume fraction fiber-reinforced epoxy systems. This form was selected because it provides the simplest continuous correction capable of reducing the tendency of the standard Halpin–Tsai model to overpredict G_12_ when fiber packing becomes dense. Accordingly, the calculation is performed in two stages for shear response: first, the uncorrected Halpin–Tsai value G_12_,HT is obtained; second, this value is multiplied by f_s_(Vf) only when Vf exceeds 0.50. For fiber contents at or below 0.50, the correction factor remains equal to unity and the original Halpin–Tsai prediction is retained.

The calibration was performed using published experimental trends for unidirectional fiber-reinforced epoxy systems at elevated fiber contents, particularly in the range Vf ≈ 0.55–0.70, where deviations from idealized micromechanical predictions become more evident due to fiber–fiber interaction and matrix-discontinuity effects [28,29]. Rather than fitting the model to a single isolated data point, the correction coefficient was selected so that the predicted G_12_ response remained consistent with the overall tendency reported in these high-volume experimental datasets. In this sense, the present calibration should be regarded as literature-informed and trend-based. The adopted value of α = 0.30 lies within the range associated with moderate reductions in matrix-mediated shear transfer at elevated fiber volume fractions. In addition, the corrected predictions were qualitatively checked against literature-reported experimental and numerical results available for Vf > 0.60, indicating that the shear-corrected formulation follows the expected saturation trend more closely than the uncorrected Halpin–Tsai relation in the high-fiber-content regime. Thus, the adopted calibration strategy was trend-based rather than point-based, with the objective of preserving the general physical response over the investigated high-Vf interval.

The correction was applied only to G_12_ and not to E_2_ because the in-plane shear modulus is more sensitive to local matrix continuity, interfacial stress transfer, and fiber clustering than the transverse Young’s modulus. In dense unidirectional packings, shear deformation depends strongly on the integrity of the resin-rich paths between adjacent fibers; therefore, small microstructural irregularities can significantly alter G_12_. By contrast, E_2_ is generally less sensitive to these local shear transfer disruptions and is more stably represented by the conventional Halpin–Tsai relation over the investigated fiber-volume-fraction range. For this reason, no additional empirical correction was imposed on E_2_ in order to avoid unnecessary over-parameterization of the model.

**Figure 1:**
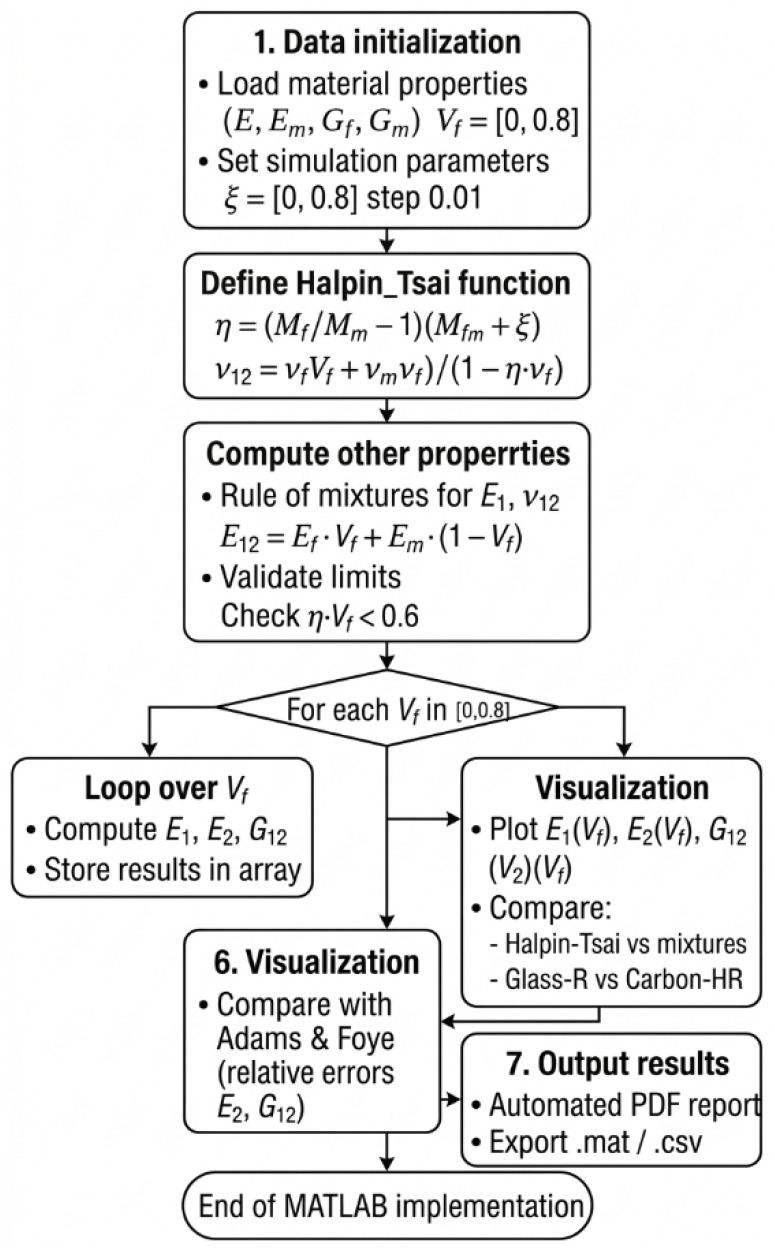
Schematic representation of the micromechanical modeling framework combining the rule of mixtures and the Halpin–Tsai equations.

The algorithm iterates over all prescribed fiber fractions, successively calling the mixture-rule and Halpin–Tsai modules. After the application of the optional shear correction, the computed elastic properties are stored in pre-allocated arrays. Final results are presented using high-resolution comparative plots, highlighting the differences between classical analytical predictions and semi-empirical estimations. Separate graphs for glass fiber and carbon fiber composites demonstrate the influence of stiffness contrast, geometry, and microstructural effects on the overall elastic response, thereby supporting both material selection and subsequent comparison with literature-reported trends [34,35]. This literature-based calibration and assessment are particularly relevant near the upper investigated range (Vf = 0.60–0.80), where the standard Halpin–Tsai formulation may become increasingly optimistic due to dense fiber packing effects. Figure 1 illustrates the overall micromechanical modeling framework adopted in this study, highlighting the integration of the rule of mixtures with the Halpin–Tsai formulation. In summary, for each Vf increment, the algorithm follows this sequence: input constituent properties → calculate E_1_ and ν12 by the rule of mixtures → calculate E_2_ and G_12_ by the Halpin–Tsai model → apply the shear correction to G_12_ when Vf > 0.50 → store the results for plotting and comparison.

## 3. Results and Discussions

We carried out a detailed analysis of the mechanical behavior of fiber-reinforced composite materials. Our focus is on evaluating the influence of fiber volume fraction (Vf) on key mechanical properties, including the longitudinal Young’s modulus (E_1_), transverse Young’s modulus (E_2_), shear modulus (G_12_), and Poisson’s ratio (ν_12_).

To achieve this, we adopt both the rule of mixtures and the Halpin-Tsai model, which are widely recognized for predicting the effective mechanical properties of composite systems based on the properties of their constituents and fiber distribution [21,23]. Overall, the predicted trends are consistent with the general behavior reported in existing composite micromechanical studies, particularly the increase in E_1_, E_2_, and G_12_ with increasing Vf and the emergence of saturation effects at elevated fiber contents. The present results were therefore interpreted in relation to representative trends available in the literature, rather than as stand-alone validated measurements. In addition, these predicted tendencies are in qualitative agreement with experimental results reported in the literature for unidirectional glass fiber/epoxy and carbon fiber/epoxy composites, particularly with respect to the progressive stiffness increase at low-to-moderate fiber contents and the reduced rate of improvement at higher Vf values. Accordingly, the discussion below emphasizes comparative trend consistency and design-oriented interpretation within the limitations of an analytical micromechanical framework.

This interpretation is supported by representative literature data reported for unidirectional fiber/epoxy composites. For example, Hamed et al. reported experimental transverse modulus values of 5.40 GPa for glass/epoxy composites at Vf = 0.476 V_f = 0.476 Vf = 0.476 and 5.718 GPa for carbon/epoxy composites at Vf = 0.545 V_f = 0.545 Vf = 0.545, together with corresponding in-plane shear modulus values of 4.085 GPa and 4.346 GPa, respectively [36]. These values are consistent with the general magnitude of the present predictions and support the expected stiffness advantage of carbon/epoxy over glass/epoxy systems. In addition, previous studies on unidirectional glass/epoxy laminates have shown that transverse response is strongly influenced by matrix continuity and fiber/matrix interfacial conditions [37], while recent experimental characterization studies on CFRP have further confirmed the importance of E2E_2E2 and G12G_{12}G12 in evaluating anisotropic elastic behavior and in supporting homogenization-based material assessment [38]. Therefore, the present results may be considered consistent with the main trends and order of magnitude reported in the literature for unidirectional fiber-reinforced epoxy composites.

### 3.1. Influence of Fiber Volume Fraction on the Mechanical Properties of Glass Fiber-Reinforced Composites

**Figure 2:**
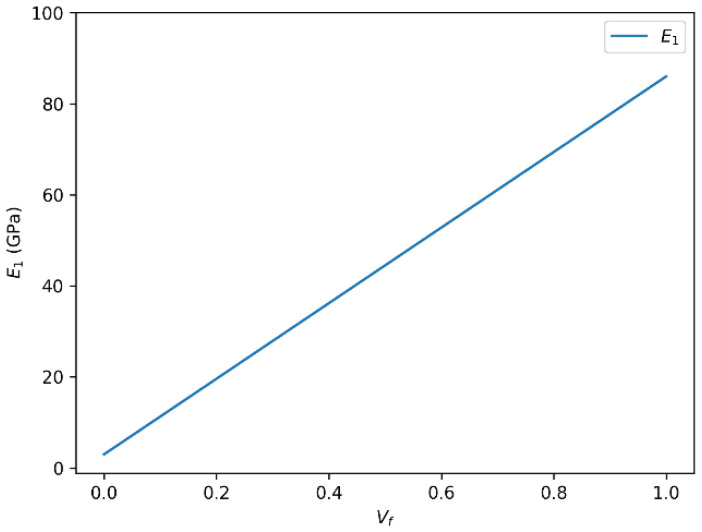
Variation in longitudinal Young’s modulus (E_1_) as a function of fiber volume fraction (Vf) for glass fiber-reinforced epoxy composites.

**Figure 3:**
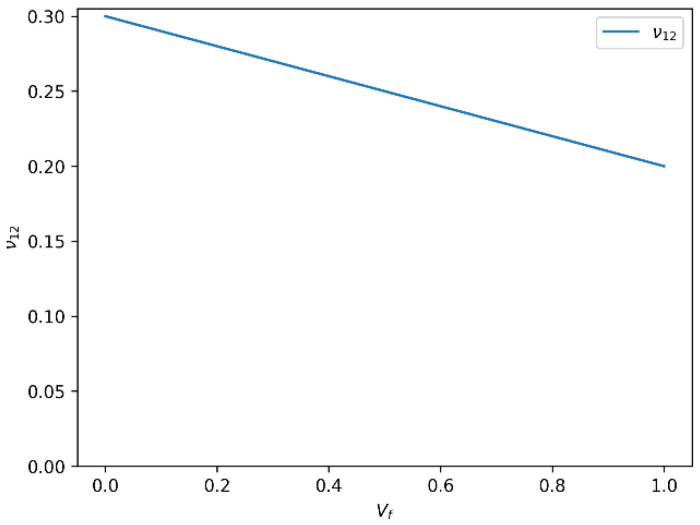
Variation in longitudinal Poisson’s ratio (ν_12_) as a function of fiber volume fraction (Vf) for glass fiber-reinforced epoxy composites.

In our analysis of glass fiber-reinforced composite materials, we identified two principal trends that describe how mechanical properties evolve with varying fiber volume fraction (Vf). These trends are critical for understanding and predicting the composite’s performance under mechanical loading. The results are influenced by multiple factors including fiber–matrix adhesion, fiber orientation, distribution uniformity, and the inherent mechanical properties of both constituents. The variation in the longitudinal Young’s modulus (E_1_) and the major Poisson’s ratio (ν_12_) of glass fiber-reinforced epoxy composites as a function of fiber volume fraction is presented in Figure 2 and Figure 3, respectively.

We observe a direct correlation between the longitudinal Young’s modulus (E_1_) and the fiber volume fraction (Vf). As the fiber content increases, E_1_ tends to rise proportionally, particularly in the low to moderate Vf range. This trend supports the fundamental concept of load transfer in composite materials: the higher the number of stiff fibers embedded in the matrix, the more effectively the load is carried along the fiber direction, enhancing stiffness and overall mechanical performance.

However, this correlation is not strictly linear across the entire range of Vf. At high fiber contents, the mechanical gains may plateau or even decline. This behavior is commonly interpreted as being associated with matrix saturation, poor wetting of fibers, fiber agglomeration, and the development of voids or interfacial defects, which compromise the stress transfer efficiency.

An inverse relationship is noted between the longitudinal Poisson’s ratio (ν_12_) and the fiber volume fraction. As Vf increases, ν_12_ generally decreases. This behavior reflects the composite’s growing resistance to transverse deformation due to the increasing stiffness in the fiber direction. Essentially, the composite becomes less prone to lateral strain under axial loading.

Similar to the trend observed for E_1_, the ν_12_-Vf relationship is not perfectly linear. It is sensitive to several factors such as fiber aspect ratio, matrix ductility, fiber distribution, and interfacial bonding quality. These elements can affect the extent to which the matrix accommodates transverse deformation, particularly at higher fiber loadings.

**Figure 4:**
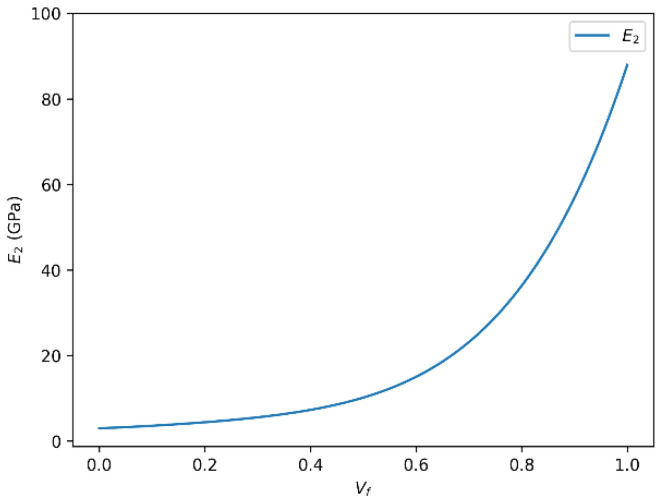
Transverse Young’s modulus (E_2_) versus fiber volume fraction (Vf) for glass fiber-reinforced epoxy composites predicted using the rule of mixtures.

**Figure 5:**
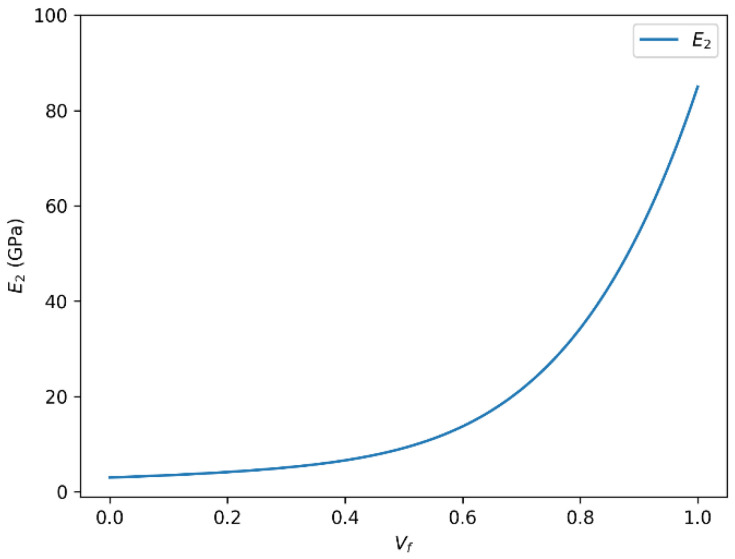
Transverse Young’s modulus (E_2_) versus fiber volume fraction (Vf) for glass fiber-reinforced epoxy composites predicted using the Halpin–Tsai model.

The mechanical performance of glass fiber-reinforced polymer (GFRP) composites is significantly influenced by the fiber volume fraction (Vf), which governs the stiffness, strength, and overall behavior of the material. Our analysis highlights several key observations regarding the impact of Vf on the transverse mechanical properties of these composites. Figure 4 and Figure 5 present the evolution of the transverse Young’s modulus (E_2_) of glass fiber-reinforced epoxy composites as a function of fiber volume fraction, as predicted by the rule of mixtures and the Halpin–Tsai model, respectively.

#### 3.1.1. Correlation Between Transverse Young’s Modulus (E_2_) and Fiber Volume Fraction (Vf)

There is a clear positive correlation between the transverse Young’s modulus (E_2_) and the fiber volume fraction (Vf). As Vf increases, E_2_ tends to rise accordingly, reflecting enhanced stiffness in the direction perpendicular to the fibers. This trend is consistent with the principles of composite reinforcement, whereby a higher fiber content strengthens the composite by restricting matrix deformation.

Our calculations show that for a specific glass fiber system, E_2_ can reach values close to 86 GPa when the fiber volume fraction approaches its optimal level. However, this increase is not strictly linear across all ranges. At higher Vf values, phenomena such as matrix saturation, fiber agglomeration, and interfacial debonding may occur, leading to diminished efficiency in load transfer and potential initiation of microstructural defects. These factors can limit further improvement in mechanical performance and may even result in a slight decline in E_2_ beyond a certain threshold.

#### 3.1.2. Role of the Halpin–Tsai Equation in Predicting E_2_

To model the relationship between E_2_ and Vf more accurately, we also apply the Halpin–Tsai equation, a semi-empirical model widely used for characterizing the mechanical behavior of composite materials. This model incorporates fiber geometry, orientation, and aspect ratio, allowing for more realistic estimations of the composite’s effective modulus.

The Halpin–Tsai model predicts the increasing trend of E_2_ with higher Vf, but it also suggests the nonlinear behavior reported in the literature. At elevated Vf, the modulus tends to plateau or slightly decrease, which may be associated with mechanical limitations due to processing-induced inhomogeneities, imperfect fiber dispersion, or reduced matrix continuity.

These modeling results suggest that the Halpin–Tsai equation is a valuable tool in the predictive analysis and optimization of GFRP composites, especially when precise control over mechanical performance is required in engineering applications. This behavior is also compatible with literature-reported experimental observations, in which the transverse stiffness of GFRP systems generally increases with fiber loading but shows reduced sensitivity at higher fiber volume fractions due to microstructural and processing-related limitations.

#### 3.1.3. Critical Observations and Design Implications

The findings underscore the critical influence of Vf on the transverse mechanical properties of fiber-reinforced composites. Accurate control and optimization of the fiber content are essential during the design and manufacturing stages to ensure that the desired performance characteristics are achieved without compromising structural integrity. For the glass fiber/epoxy system, the results indicate that the onset of transverse stiffness saturation occurs at approximately Vf ≈ 0.60, beyond which the rate of increase in E2 becomes noticeably smaller. From a design perspective, this suggests that increasing the fiber content above this level may lead to diminishing returns in transverse stiffness while simultaneously increasing the risk of manufacturing-induced defects such as poor wetting, local clustering, and reduced matrix continuity.

### 3.2. Influence of Fiber Volume Fraction (Vf) on the Shear Modulus (G12) in Glass Fiber-Reinforced Composites

**Figure 6:**
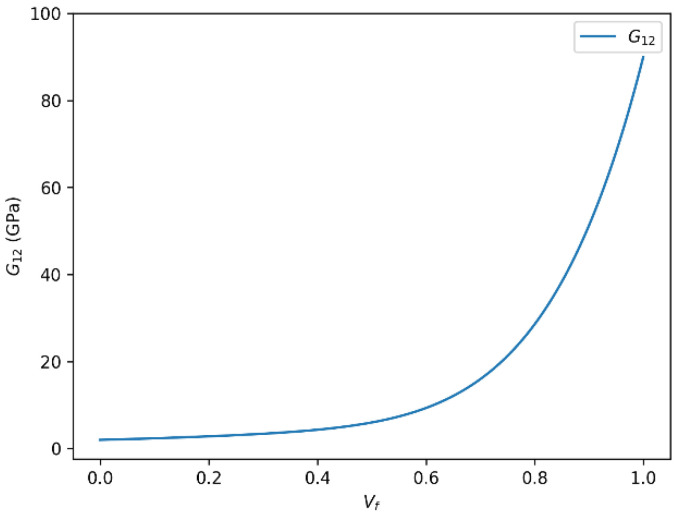
In-plane shear modulus (G_12_) as a function of fiber volume fraction (Vf) for glass fiber-reinforced epoxy composites predicted using the rule of mixtures.

**Figure 7:**
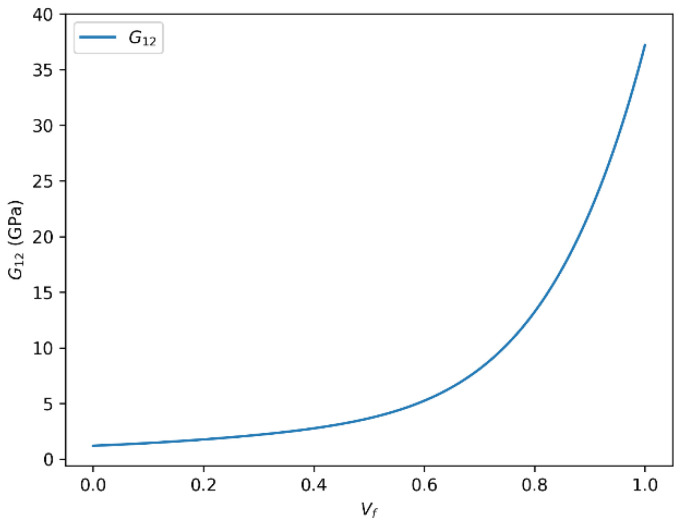
In-plane shear modulus (G_12_) as a function of fiber volume fraction (Vf) for glass fiber-reinforced epoxy composites predicted using the Halpin–Tsai model.

The dependence of the in-plane shear modulus (G_12_) on fiber volume fraction for glass fiber-reinforced composites, as predicted by the rule of mixtures and the Halpin–Tsai model, is illustrated in Figure 6 and Figure 7. We identify two main observations regarding the relationship between the in-plane shear modulus (G12) and the fiber volume fraction (Vf) in glass fiber-reinforced composites, based on analytical modeling using both the rule of mixtures and the Halpin–Tsai equation.

#### 3.2.1. Positive Correlation Between G12 and Vf

A clear and consistent correlation is observed between G_12_ and Vf, indicating that the shear modulus increases with higher fiber content. This trend is particularly evident when employing the rule of mixtures or the semi-empirical Halpin–Tsai model [19,23]. For instance, the shear modulus increases up to approximately 35.83 GPa or 35.84 GPa for a given Vf, demonstrating the reinforcing effect of glass fibers embedded within the polymer matrix. This behavior is generally associated with the superior stiffness and load-transfer capability of glass fibers, which enhance the composite’s ability to resist shear deformation.

Such enhancement in shear properties is influenced by multiple factors, including the intrinsic stiffness of the fiber, the interfacial bonding quality between fiber and matrix, and the geometric distribution of fibers. As Vf increases, the composite system becomes more fiber-dominated, thereby exhibiting improved mechanical response under shear loading.

#### 3.2.2. Nonlinear Behavior and Saturation Effects

Despite the general positive correlation, the relationship between G_12_ and Vf is not perfectly linear throughout the entire range of fiber content. At higher values of Vf, the increase in shear modulus begins to plateau, indicating a saturation effect. This nonlinear behavior is a common feature in fiber-reinforced composites, where excessive fiber loading may lead to suboptimal fiber dispersion, increased matrix–fiber interaction constraints, or even void formation factors that can limit further mechanical gains. For the glass fiber/epoxy composite, the onset of shear stiffness saturation is observed at approximately Vf ≈ 0.60, after which additional increases in fiber content produce only limited gains in G12. This result is important for material optimization because it indicates that moderate-to-high fiber contents may be more efficient than extreme fiber packing when the design objective is to improve in-plane shear performance without unnecessarily complicating processing. This saturation tendency is also consistent with literature-reported observations for high volume fraction unidirectional composites, for which the uncorrected Halpin–Tsai model may slightly overestimate shear stiffness once Vf exceeds about 0.60. Accordingly, the applied shear correction improves the physical realism of the predicted G_12_ response in the dense-packing regime. From a practical modeling perspective, the present framework is therefore expected to be most reliable within the fiber-volume-fraction range commonly achievable in manufacturing, whereas predictions at very high Vf should be interpreted more cautiously due to the increasing likelihood of defects such as poor wetting, fiber agglomeration, and void formation. Future extensions may incorporate a Vf-dependent defect factor to better represent these microstructural limitations. Moreover, the predicted saturation trend is in qualitative agreement with experimental studies on GFRP systems, where the increase in shear stiffness becomes less pronounced once dense fiber packing starts to affect matrix continuity and interfacial shear transfer.

### 3.3. Influence of Fiber Volume Fraction (Vf) on the Longitudinal Young’s Modulus (E1) and Poisson’s Ratio (ν12) in HR Carbon Fiber-Reinforced Composites

In this section, we investigate the effect of fiber volume fraction (Vf) on two key mechanical properties of high-resistance (HR) carbon fiber-reinforced composites: the longitudinal Young’s modulus (E1) and the longitudinal Poisson’s ratio (ν12). These parameters play a critical role in defining the stiffness and deformation behavior of composite materials under axial loading.

#### 3.3.1. Correlation Between Longitudinal Young’s Modulus (E1) and Fiber Volume Fraction (Vf)

**Figure 8:**
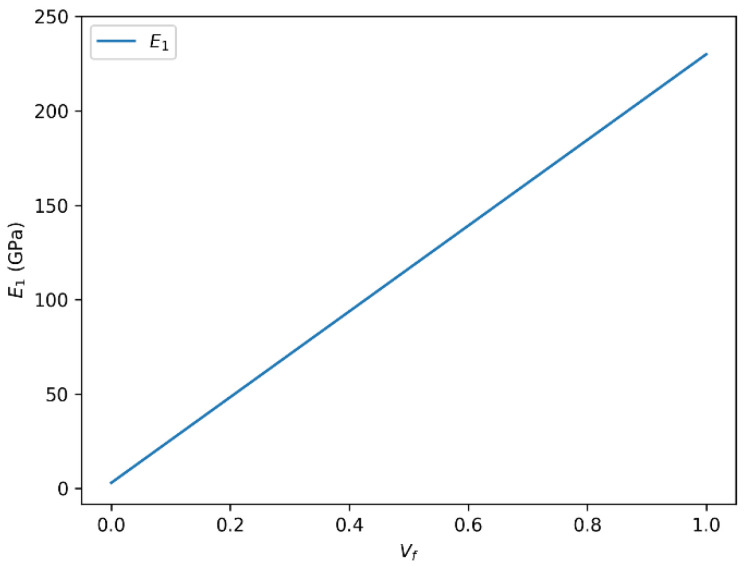
Variation in longitudinal Young’s modulus (E_1_) with fiber volume fraction (Vf) for HR carbon fiber-reinforced epoxy composites.

Figure 8 shows the variation in the longitudinal Young’s modulus (E_1_) with fiber volume fraction for HR carbon fiber-reinforced epoxy composites. We observe a strong positive correlation between E_1_ and Vf. As the fiber volume fraction increases, the longitudinal Young’s modulus also rises, reaching values up to approximately 230 GPa for high Vf levels. This trend is consistent with the classical theory of composite reinforcement, where increasing the proportion of stiff fibers within the matrix enhances axial stiffness.

However, this relationship is not strictly linear across the entire range of Vf. Beyond a certain threshold, the reinforcing effect tends to plateau or even diminish. Several factors may influence this behavior:

- Matrix Saturation: At high Vf, the polymer matrix may become saturated, limiting its ability to properly wet and bond with the fibers.
- Fiber Packing and Dispersion: Excessive fiber content can lead to poor dispersion or fiber misalignment, which negatively impacts load transfer.
- Fiber–Matrix Interface Quality: At elevated Vf, the interface between fibers and matrix may become more susceptible to defects, reducing overall composite integrity.

These considerations highlight the importance of optimizing Vf to balance mechanical performance and material processability.

#### 3.3.2. Stability of Longitudinal Poisson’s Ratio (ν12) with Increasing Vf

**Figure 9:**
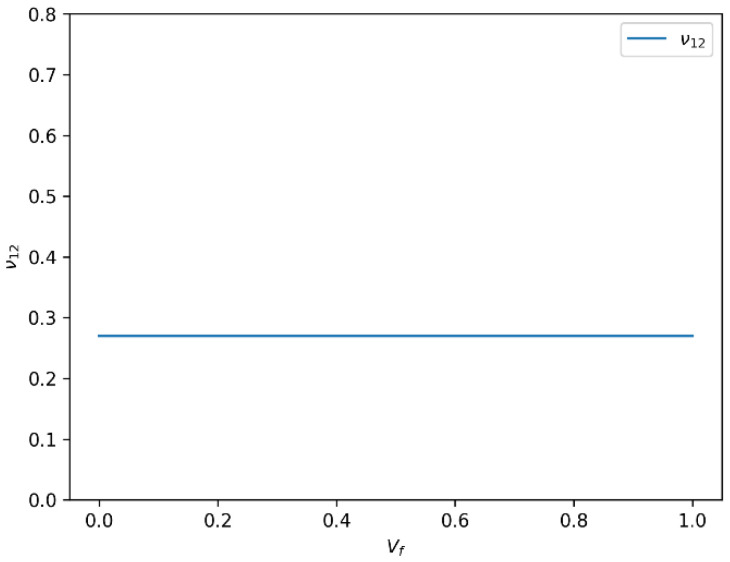
Variation in longitudinal Poisson’s ratio (ν_12_) with fiber volume fraction (Vf) for HR carbon fiber-reinforced epoxy composites.

The evolution of the longitudinal Poisson’s ratio (ν_12_) as a function of fiber volume fraction for HR carbon fiber composites is presented in Figure 9. In contrast to the behavior of E1, the longitudinal Poisson’s ratio (ν12) remains remarkably stable with increasing Vf, maintaining a value close to 0.3 across the studied range. This indicates that, although the stiffness in the fiber direction increases, the ratio of transverse to axial strain remains nearly constant. This stability reflects the dominant influence of the fiber phase in the longitudinal direction and the relatively minor role of fiber volume fraction on transverse strain response.

### 3.4. Influence of Fiber Volume Fraction on Transverse Young’s Modulus (E_2_) in HR Carbon Fiber Composites

**Figure 10:**
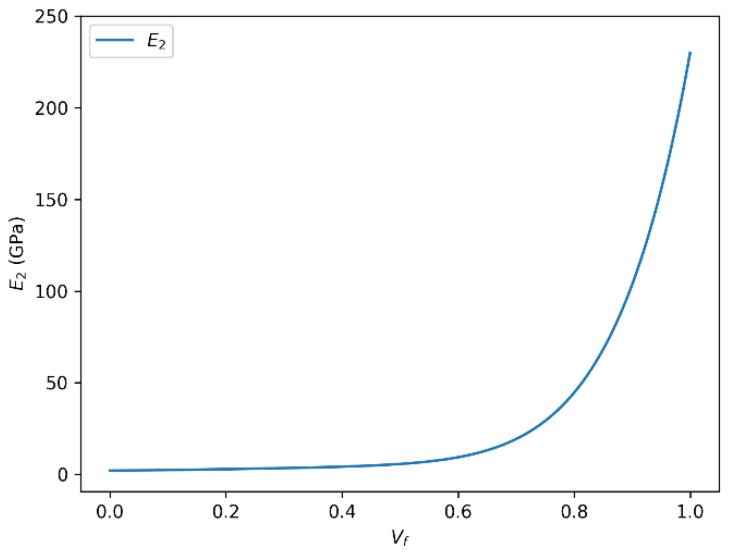
Transverse Young’s modulus (E_2_) as a function of fiber volume fraction (Vf) for HR carbon fiber-reinforced epoxy composites predicted using the rule of mixtures.

**Figure 11:**
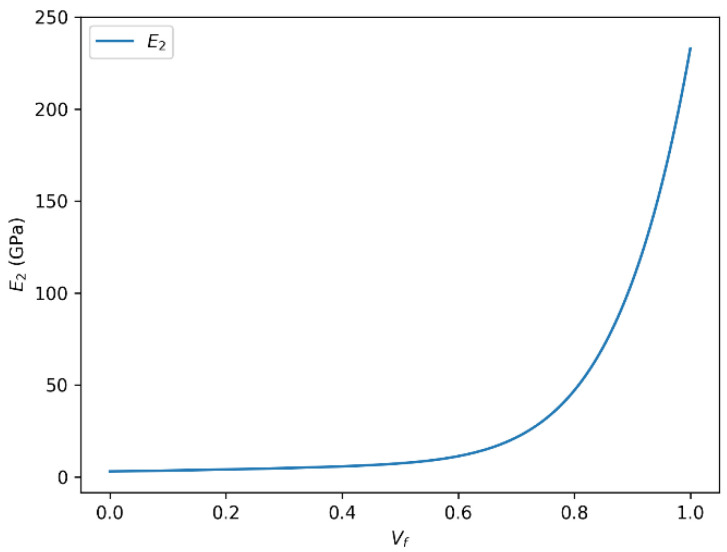
Transverse Young’s modulus (E_2_) as a function of fiber volume fraction (Vf) for HR carbon fiber-reinforced epoxy composites predicted using the Halpin–Tsai model.

Figure 10 and Figure 11 depict the variation in the transverse Young’s modulus (E_2_) with fiber volume fraction for HR carbon fiber-reinforced epoxy composites, as predicted by the rule of mixtures and the Halpin–Tsai model, respectively. The two descriptions below illustrate the evolution of the transverse Young’s modulus (E_2_) as a function of the fiber volume fraction (Vf), calculated respectively by the rule of mixtures and the Halpin–Tsai equation.

In both cases, a strong positive correlation is observed between the transverse Young’s modulus and the fiber volume fraction. As the fiber volume fraction increases, the transverse Young’s modulus (E_2_) increases accordingly and asymptotically approaches a theoretical upper bound governed by the intrinsic stiffness of the fiber phase, rather than representing a physically attainable transverse modulus. It should be emphasized that E_2_ remains significantly lower than the longitudinal fiber modulus in physically realizable composite systems due to transverse load transfer limitations and matrix-dominated deformation mechanisms.

It is important to note that this relationship is not strictly linear over the entire range of fiber volume fractions. There exists a saturation effect, where beyond a certain Vf threshold, the reinforcing influence of the fibers plateaus or may even slightly diminish. This plateau corresponds to the physical limits of the fiber–matrix interaction and the load transfer efficiency in the composite structure.

The observed maximum transverse Young’s modulus (~230 GPa) at Vf = 1 highlights the performance limit governed by the intrinsic properties of the carbon fibers themselves. Factors such as fiber matrix adhesion, fiber distribution uniformity, and matrix properties also critically influence the effective modulus and the overall mechanical behavior of the composite material. Based on the predicted trend, the onset of transverse stiffness saturation for the HR carbon fiber/epoxy system occurs at approximately Vf ≈ 0.65, where the slope of the E2-Vf curve becomes significantly less pronounced. This indicates that the carbon fiber system can sustain useful stiffness gains over a slightly broader fiber-volume-fraction range than the glass fiber system, although the benefit also diminishes at high Vf.

### 3.5. Influence of Fiber Volume Fraction on Shear Modulus (G12) in HR Carbon Fiber Composites

**Figure 12:**
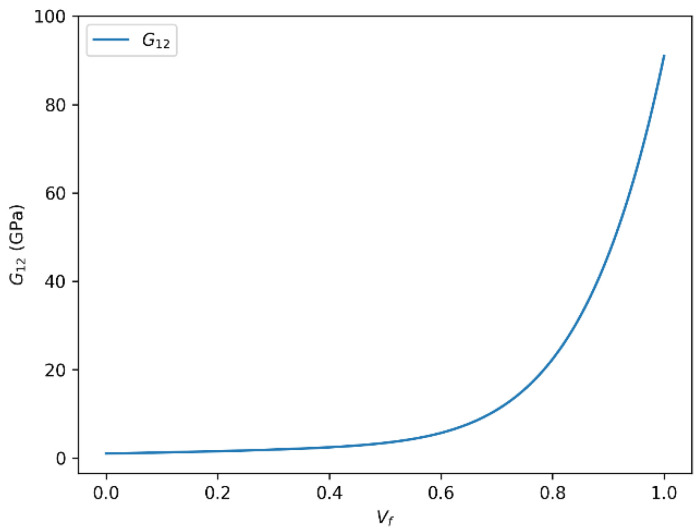
In-plane shear modulus (G_12_) versus fiber volume fraction (Vf) for HR carbon fiber-reinforced epoxy composites predicted using the rule of mixtures.

**Figure 13:**
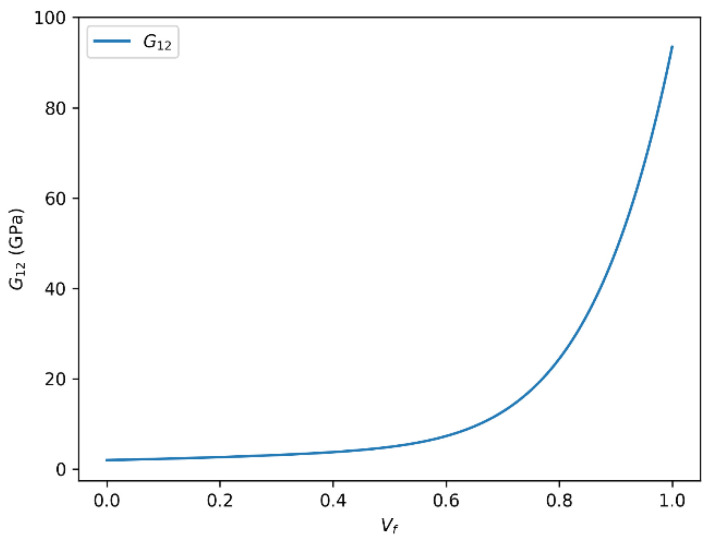
In-plane shear modulus (G_12_) versus fiber volume fraction (Vf) for HR carbon fiber-reinforced epoxy composites predicted using the Halpin–Tsai model.

The relationship between fiber volume fraction and the in-plane shear modulus (G_12_) for HR carbon fiber composites, obtained using both predictive approaches, is illustrated in Figure 12 and Figure 13. The two presented approaches detail how the shear modulus (G_12_) varies as a function of the fiber volume fraction (Vf) in HR carbon fiber composites: one using the rule of mixtures and the other based on the Halpin–Tsai equation. In both cases, a clear positive correlation is observed between the shear modulus and the fiber volume fraction. Specifically, as Vf increases, the shear modulus (G_12_) correspondingly increases, reaching an asymptotic limit of approximately 86.46 GPa with the rule of mixtures and about 86.56 GPa with the Halpin–Tsai model when Vf approaches unity.

This trend is consistent with fundamental composite reinforcement principles, where increasing the fiber content enhances load transfer efficiency, thereby improving mechanical properties such as shear resistance and overall stiffness. The underlying mechanisms include improved fiber–matrix interaction and stress distribution within the composite microstructure.

It should be noted, however, that the relationship between G_12_ and Vf is not strictly linear over the entire volume fraction range. Nonlinear effects arise due to factors such as fiber packing limitations, matrix saturation, and potential fiber–fiber interactions at high volume fractions. These effects contribute to a plateau or saturation behavior, indicating diminishing returns on shear modulus improvements beyond a critical fiber content.

The observed saturation shear modulus values around 86.46 GPa (rule of mixtures) and 86.56 GPa (Halpin–Tsai) highlight the intrinsic material limits governed by fiber and matrix properties as well as composite architecture. These insights are crucial for the accurate modeling and optimization of fiber-reinforced composites, particularly in applications demanding tailored mechanical performance and reliability. For HR carbon fiber/epoxy composites, the onset of shear stiffness saturation is observed at approximately Vf ≈ 0.65. Beyond this range, the additional increase in G12 becomes progressively smaller, indicating that the efficiency of further fiber addition decreases despite the higher absolute stiffness level of the carbon-reinforced system. This trend is in qualitative agreement with literature-based high Vf composite data and supports the use of the shear-corrected Halpin–Tsai formulation at elevated fiber contents, where the standard form may otherwise remain slightly non-conservative in predicting G_12_ evolution. A similar tendency has been reported in experimental carbon fiber/epoxy studies, where the shear response continues to increase with fiber content but departs from idealized linear growth at high Vf because of packing and interface-related effects.

### 3.6. Comparative Analysis of Mechanical Properties: Carbon-HR vs. Glass-R Reinforced Composites

The following comparative analysis investigates the evolution of mechanical properties in composite materials reinforced with two distinct types of fibers: Carbon-HR and Glass-R. The key mechanical indicators considered include longitudinal and transverse Young’s modulus (E_1_ and E_2_), shear modulus (G_12_), and Poisson’s ratio (ν_12_), all as functions of the fiber volume fraction (Vf). The performance disparities are explained based on intrinsic material properties, load transfer efficiency, interfacial bonding, and stiffness-to-weight ratio factors that significantly influence the mechanical behavior of fiber-reinforced composites. A direct comparison of the predicted values also indicates that at low, medium, and high fiber contents, Carbon-HR consistently provides higher E_1_, E_2_, and G_12_ values than Glass-R. This difference becomes more pronounced as Vf increases and is consistent with the higher stiffness contrast parameter (η) associated with the carbon fiber system in the Halpin–Tsai formulation, which leads to a stronger reinforcing effect in both the transverse and shear responses.

#### 3.6.1. Longitudinal Young’s Modulus (E_1_) Evolution with Fiber Volume Fraction (Vf) Longitudinal Young’s Modulus (E_1_) Analysis

**Figure 14:**
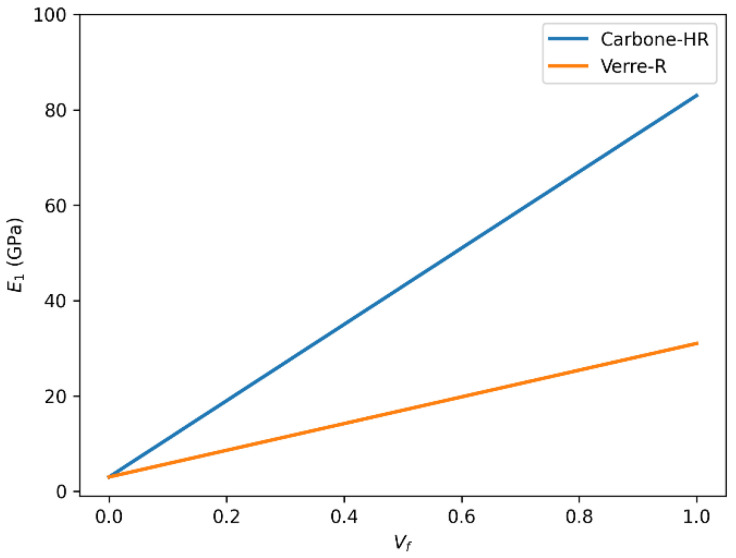
Comparison of longitudinal Young’s modulus (E_1_) as a function of fiber volume fraction (Vf) for Glass-R and HR carbon fiber-reinforced epoxy composites.

Figure 14 compares the evolution of the longitudinal Young’s modulus (E_1_) for Glass-R and HR carbon fiber-reinforced composites as a function of fiber volume fraction. As illustrated in Figure 14, the longitudinal modulus E_1_ increases for both Carbon-HR and Glass-R composites with rising fiber volume fraction Vf. However, the maximum value of E_1_ for Carbon-HR approaches approximately 230 GPa, which corresponds to the intrinsic Young’s modulus of the carbon fibers, while that of Glass-R saturates around 86 GPa. This large difference is attributed to the higher intrinsic stiffness of carbon fibers, their superior aspect ratio, and improved stress transfer capability through the matrix.

This finding indicates that Carbon-HR provides a significantly stiffer reinforcement in the fiber direction, making it suitable for high-performance structural applications requiring minimal axial deformation.

#### 3.6.2. Longitudinal Poisson’s Ratio (ν_12_) Variation with Fiber Volume Fraction (Vf)

**Figure 15:**
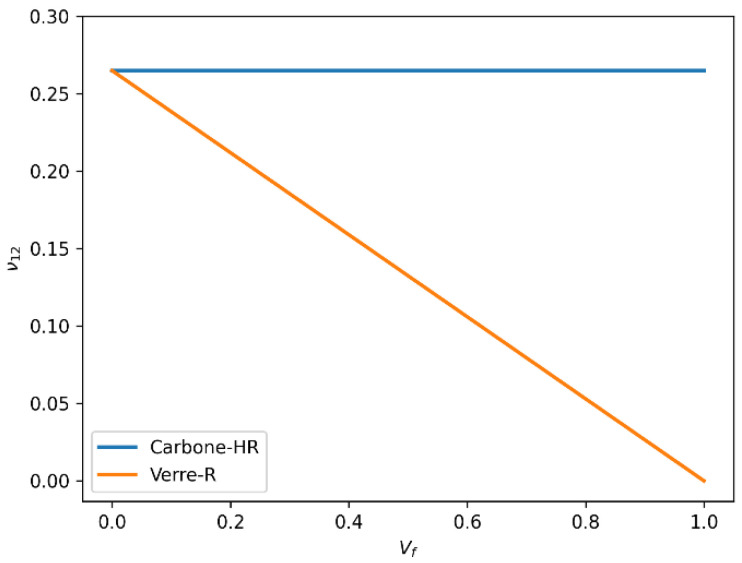
Comparison of longitudinal Poisson’s ratio (ν_12_) as a function of fiber volume fraction (Vf) for Glass-R and HR carbon fiber-reinforced epoxy composites.

The variation in the longitudinal Poisson’s ratio (ν_12_) for both Glass-R and HR carbon fiber composites with increasing fiber volume fraction is shown in Figure 15. In terms of longitudinal Poisson’s ratio, ν_12_, the results show that Glass-R exhibits a decreasing trend from 0.3 to 0.2 as Vf increases, while Carbon-HR remains approximately constant at 0.3. This stability suggests a more isotropic lateral deformation in Carbon-HR composites and indicates better interfacial bonding, which limits transverse strain under longitudinal loading.

A lower Poisson’s ratio generally implies improved dimensional stability and reduced transverse deformation, which is advantageous in applications subjected to complex loading.

#### 3.6.3. Transverse Young’s Modulus (E_2_) According to Volume Fraction and Predictive Models

**Figure 16:**
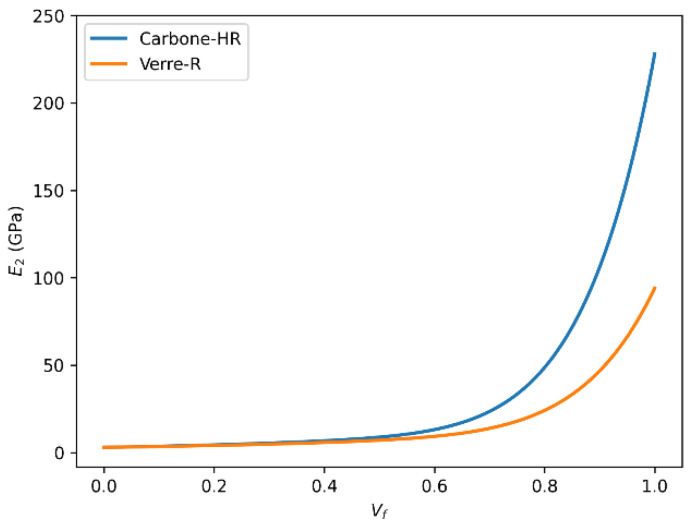
Comparison of transverse Young’s modulus (E_2_) as a function of fiber volume fraction (Vf) for Glass-R and HR carbon fiber-reinforced epoxy composites predicted using the rule of mixtures.

**Figure 17:**
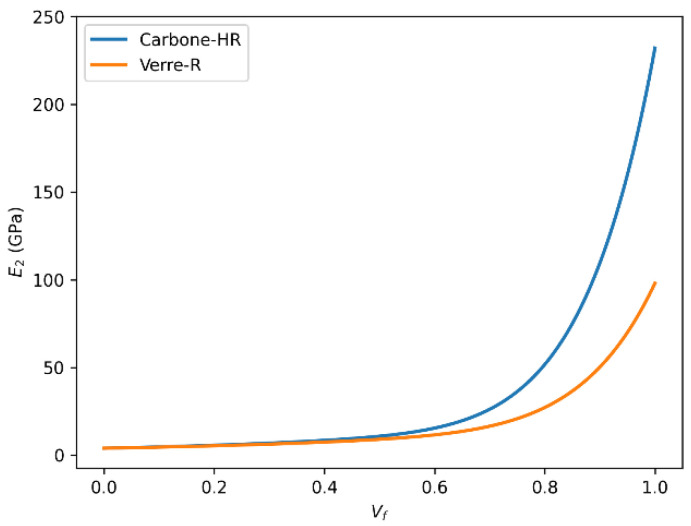
Comparison of transverse Young’s modulus (E_2_) as a function of fiber volume fraction (Vf) for Glass-R and HR carbon fiber-reinforced epoxy composites predicted using the Halpin–Tsai model.

Figure 16 and Figure 17 present a comparative evaluation of the transverse Young’s modulus (E_2_) of Glass-R and HR carbon fiber-reinforced composites as a function of fiber volume fraction using the rule of mixtures and the Halpin–Tsai model. The transverse modulus E_2_ reflects the stiffness perpendicular to the fiber direction. Both estimation methods, rule of mixtures and Halpin–Tsai, reveal increasing E_2_ with rising Vf. For Carbon-HR, the modulus reaches a peak of around 22 GPa, compared to about 10 GPa for Glass-R.

This difference is influenced not only by the higher modulus of carbon fibers but also by their anisotropy and compatibility with the matrix, which enhance load transfer in off-axis directions.

The Halpin–Tsai model, which incorporates shape and orientation factors, provides a more accurate prediction of E_2_, especially at moderate to high Vf levels.

#### 3.6.4. Shear Modulus (G_12_) Evolution with Fiber Volume Fraction (Vf)

**Figure 18:**
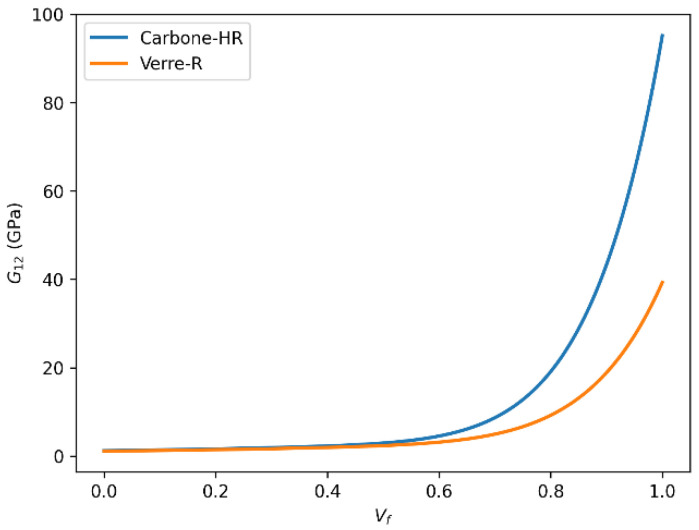
Comparison of in-plane shear modulus (G_12_) as a function of fiber volume fraction (Vf) for Glass-R and HR carbon fiber-reinforced epoxy composites predicted using the rule of mixtures.

**Figure 19:**
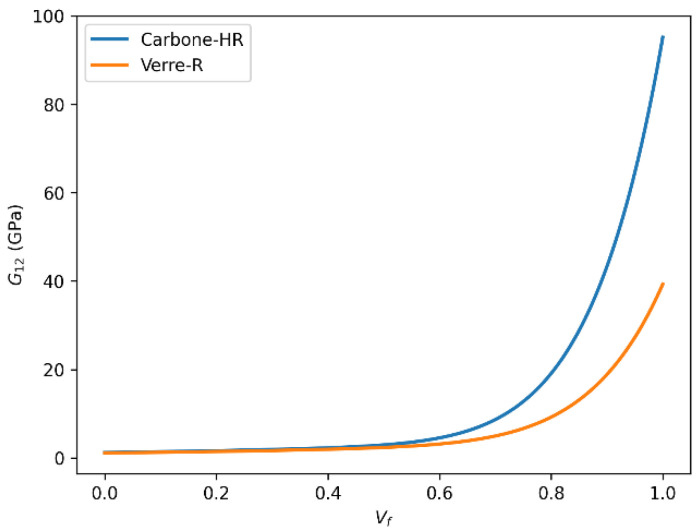
Comparison of in-plane shear modulus (G_12_) as a function of fiber volume fraction (Vf) for Glass-R and HR carbon fiber-reinforced epoxy composites predicted using the Halpin–Tsai model.

The comparative evolution of the in-plane shear modulus (G_12_) for Glass-R and HR carbon fiber-reinforced epoxy composites with increasing fiber volume fraction is illustrated in Figure 18 and Figure 19. Shear modulus G_12_ quantifies resistance to in-plane shear deformation. The data show that both Carbon-HR and Glass-R composites exhibit increasing G_12_ with Vf, but Carbon-HR reaches a significantly higher limit of 86 GPa, compared to 35 GPa for Glass-R.

This enhanced shear resistance in Carbon-HR composites is associated with the inherent rigidity of carbon fibers, better alignment, and reduced interfacial shear lag due to strong adhesion with the matrix. The mechanical performance of fiber-reinforced composites is highly dependent on the type of fiber used, the fiber volume fraction, and the fiber–matrix interfacial properties. Carbon-HR fibers consistently outperform Glass-R fibers in terms of stiffness (E_1_, E_2_), shear resistance (G_12_), and dimensional stability (ν_12_), particularly at higher Vf values. These findings underscore the importance of selecting appropriate reinforcements based on the target mechanical performance, structural efficiency, and cost–performance tradeoffs in composite design. From a practical design standpoint, the results indicate that carbon fiber/epoxy composites are preferable for stiffness-critical applications, whereas glass fiber/epoxy systems may remain attractive where cost-efficiency and processability are more important. In both systems, the observed saturation behavior beyond approximately Vf = 0.60–0.65 suggests that increasing the fiber content above this interval should be justified by specific performance requirements, since the marginal stiffness gain becomes progressively smaller. Moreover, this behavior is compatible with literature-reported experimental tendencies at high fiber contents, for both glass and carbon fiber-reinforced epoxy systems, supporting the validity of the calibrated shear-response predictions used in the present analysis.

## 4. Conclusions

In this study, the elastic behavior of Glass-R and Carbon-HR fiber-reinforced epoxy composites was evaluated using a combined framework based on the rule of mixtures and the Halpin–Tsai micromechanical model. The analysis focused on the prediction of the longitudinal and transverse Young’s moduli (E_1_ and E_2_), the in-plane shear modulus (G_12_), and the major Poisson’s ratio (ν_12_) over a wide range of fiber volume fractions (Vf up to 0.80).

The results show that Carbon-HR composites exhibit consistently higher stiffness than Glass-R systems across the investigated fiber volume fraction range. At comparable fiber contents, Carbon-HR composites demonstrate approximately 20–25% higher longitudinal stiffness and 10–15% higher shear modulus, indicating their suitability for applications requiring high structural rigidity and dimensional stability.

The comparative evaluation of predictive models indicates that the Halpin–Tsai formulation provides more realistic estimates of transverse and shear elastic properties, particularly at moderate to high fiber volume fractions, whereas the rule of mixtures shows limitations for anisotropic stiffness prediction beyond the longitudinal direction. Within the scope of the present analytical study, these estimates were interpreted in relation to literature-reported composite behavior and used to support comparative, design-oriented assessment.

The results also show that the onset of stiffness saturation occurs approximately at Vf ≈ 0.60 for the glass fiber/epoxy system and at Vf ≈ 0.65 for the carbon fiber/epoxy system, particularly in the E2 and G12 responses. Beyond these ranges, additional fiber loading yields progressively smaller stiffness gains, indicating diminishing returns in relation to manufacturing complexity and potential microstructural irregularities.

From a design standpoint, the nonlinear dependence of elastic properties on fiber volume fraction highlights the need to balance stiffness enhancement with processing quality and manufacturability constraints. Accordingly, fiber volume fractions in the range of about 0.60–0.65 may be considered an efficient design window for achieving substantial stiffness improvement while avoiding excessive sensitivity to processing-related limitations. The proposed modeling framework offers a practical and efficient tool for preliminary composite design and material selection. Because the present work is based on analytical prediction, future studies should include direct experimental measurements and/or numerical homogenization to further verify the proposed high volume fraction response, particularly for the in-plane shear modulus.

## Figures and Tables

**Table 1:** Key physical and mechanical properties of the constituent materials used in the micromechanical modeling, based on representative engineering values.

Component	Density (kg/m^3^)	Young’s Modulus (GPa)	Shear Modulus (GPa)	Poisson’s Ratio	Tensile Strength (MPa)
Glass R Fiber	2500	86	—	0.20	3200
HR Carbon Fiber	1750	230	50	0.30	3200
Epoxy Resin	1200	3.4	1.42	0.20	—

## Data Availability

The original contributions presented in this study are included in the article. Further inquiries can be directed to the corresponding author.

## Nomenclature

**Symbol**	**Description**	**Unit**
E1	Longitudinal Young’s modulus	GPa
E2	Transverse Young’s modulus	GPa
G12	In plane shear modulus	GPa
ν12	Major Poisson’s ratio	-
Vf	Fiber volume fraction	-
Mf	Fiber modulus (E or G depending on context)	GPa
Mm	Matrix modulus (E or G depending on context)	GPa
η	Stiffness contrast parameter	-
ξ	Geometric factor (fiber shape and arrangement)	-
CFRP	Carbon fiber-reinforced polymer	-
UV	Ultraviolet radiation	-
